# Estimated incidence and transmission intensity of rubella infection in Zambia pre-vaccine era 2005–2016

**DOI:** 10.1017/S0950268822001868

**Published:** 2022-12-20

**Authors:** Mazyanga L. Mazaba, Samuel Bosomprah, Daniel Cohen, Mwaka Monze, Seter Siziya

**Affiliations:** 1 Michael Chilufya Sata School of Medicine, Copperbelt University, Ndola, Zambia; 2 Zambia National Public Institute, Ministry of Health, Lusaka, Zambia; 3 Department of Biostatistics, School of Public Health, University of Ghana, Legon, Accra; 4 School of Public Health, Sackler Faculty of Medicine, Tel Aviv University, Tel Aviv, Israel; 5 Virology Unit, University Teaching Hospital, Ministry of Health, Lusaka, Zambia

**Keywords:** Infectious disease epidemiology, infectious disease, modelling, outbreaks, rubella

## Abstract

The rubella disease burden in Zambia may be under-estimated. Using models, we describe the transmission dynamics, determine the incidence estimates and assess the level of underestimation of the real burden of rubella infection in Zambia during the pre-vaccination period 2005–2016. This study used both the deterministic compartmental model and likelihood-based method using a Bayesian framework to describe the epidemiology of rubella. A total of 1313 cases of rubella were confirmed with the highest annual number of 255 new cases recorded in 2008. However, 2014 recorded the highest monthly median positivity rate of 9.0%. The observed median rubella cases were 5.5. There was a seasonal pattern in the occurrence of laboratory-confirmed rubella, with higher test positivity rates of rubella infection usually recorded in the months of September, October and November. The modelled monthly median incidence of rubella infection among the general population was 76 and 20 among pregnant women. The incidence of rubella among the non-pregnant women was 44. The average effective reproductive number (Rt) between 2005 and 2016 was estimated as 1.2 with the peak of infection occurring in 2016. The measles surveillance system underestimates the observed burden of rubella. A mass vaccination campaign conducted between January and July is recommended.

## Introduction

The major impact for rubella is among pregnant women. Whilst rubella virus infection causes a mild febrile and rash disease in children and adults, infection during pregnancy can result in miscarriage, foetal death, stillbirth or birth defects [1].

Zambia has no deliberate policy on rubella surveillance, however cases of rubella are identified through routine disease surveillance systems and integrated measles/rubella case-based surveillance [2]. The estimated rubella disease burden relies on the measles surveillance programme where samples testing negative to measles immunoglobulin M (IgM) are tested for rubella IgM. A negative measles IgM sample indicates that the patient suspected of having measles has not been exposed to the measles. Considering rubella has similar symptoms, though mostly more mild, the samples are then tested for rubella IgM. A positive IgM is indicative of an active infection.

Blood or throat swab samples are collected from persons suspected of having a measles infection. A suspected measles case is defined as persons displaying fever and generalised maculopapular (non-vesicular) rash, or a patient whom a health care worker suspects has measles.

Rubella-containing vaccines have been introduced in many countries to prevent these adverse pregnancy outcomes [3] and congenital rubella syndrome (CRS). Rubella vaccine was introduced in Zambia as a measles-rubella vaccine into the national immunisation schedules in September 2016. Two doses of measles-rubella vaccine are given at a minimum interval of 9 months starting at the age of 9 months [4].

Rubella surveillance is necessary to monitor disease burden after introduction of rubella-containing vaccine [3]. Disease surveillance data may be biased partly due to non-specific diagnosis, targeted to young children and to underreporting; for example, in 2016, WHO estimated 7 million rubella cases globally [5], but only 132 137 were reported to the WHO [6]. For rubella, reporting is even less sensitive because 20–50% of rubella cases are asymptomatic [1]. The number of cases reported will depend on the quality of the system in identifying cases of rubella. However, well-conducted seroprevalence studies can help to improve the reliability of rubella incidence rates [7].

The average incidence rates between 1977 and 1985 for rubella infection in the age group 15–44 were 399, 427, 373, 206 and 465 per 100 000 pregnancies for Cote d'Ivoire in Abidjan [8], Nigeria and western Nigeria [9, 10] and Zambia, Lusaka [11], respectively. In 2009, 17388 cases of rubella with an incidence of 2.11 per 100 000 populations were reported in Africa [4].

An infection will persist in a community if the average number of secondary cases caused by an infected individual is equal to or greater than 1. If it is less than 1, then the generation of secondary cases is not adequate to maintain the infection in a community. Papadopoulos and Vynnycky [12] estimated the basic reproduction number (Ro) for rubella for 98 settings from around the world and found that Ro was <5, 5–10 and >10 for 81, 14 and 3 settings respectively. In Africa, Ro was in the range 1.5–2.0 between 1979 and 2009. Particularly, in Zambia, the estimate was for the period 1979 and 1980.

Updated reliable estimates based on modelling studies are important in tracking the rubella epidemic in designing interventions to control and eliminate it. Hence, the objective of the study was to determine the incidence of rubella infection and its rate of reproduction in Zambia in a modelling study during the pre-vaccination period 2005–2016.

## Methods

### Model formulation and analysis for rubella infection in Zambia

This study used a deterministic compartmental model to describe the transmission dynamics of rubella infection in Zambia. Compartmental models are widely used to study the dynamics of viral infections among other infections that are transmitted in populations [13]. The total population (*N*) is divided into the following epidemiological classes: susceptible, *S* (individuals who are not yet infected but may be infected with rubella over time). Some of these susceptible populations may be vaccinated or will be exposed when they come into contact with either an infectious pregnant woman or an infectious individual in the general population. The exposed (E) or latent class are individuals (pregnant women or the general population) who have been infected with rubella but are not infectious to others. Within days, this group of exposed individuals becomes infectious and move to the infectious (I) class. The infectious sub-classes are pregnant women and the general population that can transmit rubella infection to others in the susceptible class due to the high accumulation of viral load. Both the exposed and the infectious classes may recover from rubella infection at different rates and will be classified under the recovery sub-class. Infectious pregnant women can either host an infected foetus or an uninfected foetus due to the possibility of vertical transmission and the infected foetus may be either aborted or they will be born with CRS. This study emphasised that children may recover from the viral infection and join the recovery class.

The model adjusts for seasonality by [13] including seasonality-dependent force of infection *λ*(*t*) = *β*_0_ + *β*_1_sin(2*πt*/12) + *β*_2_cos (2*πt*/12) where *t* is time in months. The study distinguished between different levels of vaccination by assuming that individuals who are vaccinated only receive one dose and modelled the possibility of receiving full doses of vaccination. The transmission rate was estimated from the observed number of cases using the non-linear least square estimation via Levenberg–Marquardt algorithm that minimises the sum of squared error between the observed cumulative number of rubella cases and predicted cumulative cases. Other parameters were carefully chosen to fit our definition to fit the model. The time step for the model is monthly and we modelled counterfactual scenario of no vaccination post 2016. The systems of differential equations were analysed using the ‘*desolve*’ package in *R* [14]. Using the approach of Cori *et al*. [15], we estimated the time-dependent effective reproduction number Rt using monthly incidence time series rubella data between 2005 and 2016. The Cori *et al*. approach assumes that once infected, individuals have an infectivity profile given by a probability distribution *W*_*s*_, and is dependent on time since infection of the case, *s*, but independent of calendar time, *t*. The authors estimated the instantaneous reproductive number as the ratio of the number of new infections generated at time step *t*, *I*_*t*_, to the total infectiousness of infected individuals at time *t*, given by 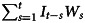, the sum of infection incidence up to time step *t*  −  1, weighted by the infectivity function *W*_*s*_. *R*_*t*_ is the average number of secondary cases that each infected individual would infect if the conditions remained as they were at time *t*. The incidence at time *t* is Poisson distributed with mean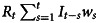.

A description of variables and parameters is given in Table 1 below.

**Table 1.** Description of variables and parameters

Variables	Definition	Estimate	Source
***S***_***T***_	The number of uninfected people in the population but are susceptible to rubella infection at time *t*	17 900 000	https://www.worldometers.info/world-population/zambia-population/
***S***_***G***_	The number of uninfected pregnant women in Zambia	5000	Initial condition
***S***_***P***_	The number of uninfected populations in Zambia	17 400 000	Initial condition
*E*_*G*_	The number of people in the general population at time *t* excluding those who are pregnant that have been exposed to rubella virus but are not yet infectious to others	20	Initial condition
*E*_*P*_	Number of pregnant women at time *t* who have been exposed to rubella virus but are not yet infectious	10	Initial condition
*I*_*G*_	Number of the infectious general population at time *t*	5	Initial condition
*I*_*P*_	Number of pregnant women at time *t* who are infectious	5	Initial condition
*V*_1_	First dose of vaccinated population	0	Initial condition
*V*_2_	Second dose of vaccinated population	0	Initial condition
*I*_*F*_	The number of rubella infected foetuses at time *t*	0	Initial condition
*R*	The number of recovered individuals at time *t*	0	Initial condition
*I*_*NF*_	The number of the non-infected foetus at time *t*	0	Initial condition
*N*	The total population	18 383 956	https://www.worldometers.info/world-population/zambia-population/
Parameters			
*π*	Per capita birth rate in Zambia	6.2 × 10^−10^	Estimated
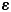	Rate of vaccination among the susceptible population in Zambia	0.00	Assumed be zero since there was no vaccination
*μ*_*R*_	Rubella disease-induced death	1.0 × 10^−10^	Estimated
***ρ***	The rate at which first dose of vaccinated population moves to the susceptible compartment	0.00	Assumed be zero since there was no vaccination
***μ***	Per capita natural mortality rate	6.2 × 10^−10^	Estimated
***λ***_***IP***_	Season-dependent force of infection among pregnant women	varies	Estimated
***λ***_***IG***_	Season-dependent force of infection among the general population	varies	Estimated
*σ*_*P*_	Progression rate from exposed class population to infectious class among the pregnant women	1.0 × 10^−9^	Estimated
*σ*_*G*_	Progression rate from exposed class to infectious class among the general population	1.0 × 10^−11^	Estimated
*γ*	Rate of recovery among those who have had single dose vaccination	0.00	Assumed be zero since there was no vaccination
*η*_*G*_	The recovery rate of the exposed general population	0.0006	Estimated
*η*_*P*_	The recovery rate of the exposed pregnant women or the general population	0.0006	Estimated
*η*_*G*_	The recovery rate of the infectious general population	0.0006	Estimated
*η*_*P*_	The recovery rate of the infectious pregnant women	0.0006	Estimated
***k***	The rate of recovery among those who received full vaccination	0.00	Assumed to be zero since there was no vaccination
*θ*	The rate of receiving 2nd dose vaccination among those who had received first dose	0.00	Assumed to be zero since there was no vaccination
***α***	Proportion of infected foetus that develop congenital rubella syndrome	0.00	Initial condition
1 − *α*	The proportion of infected foetus that do not develop congenital rubella syndrome but are aborted	1.00	Initial condition based on ***α***
*ω*	The rate of abortion in Zambia	0.00	Initial condition

**Fig. 1.**
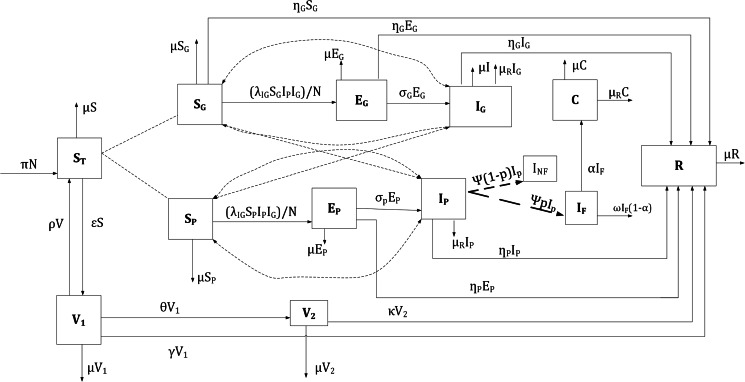
A compartmental model showing the dynamics of rubella infection in Zambia.

Figure 1 is a compartmental model showing the conceptualised dynamics of rubella between 2005 and 2016 in Zambia

### Systems of differential equations


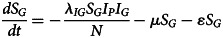


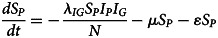


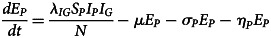


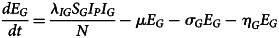


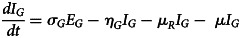


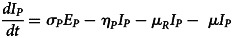


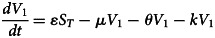


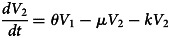


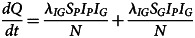


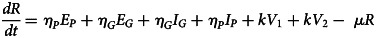


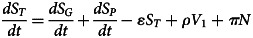


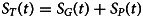


The total population size *N*(*t*) is assumed to be constant and homogenous:

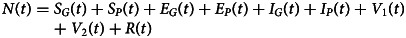


We did not provide detailed analytic expression of rubella infection since the focus of the study was to track the incidence of rubella.

### Ethical consideration

The data used in this study are de-identified secondary data from the Government-run measles surveillance programme. Ethical clearance was sought from the Ministry of Health, ERES Coverage IRB (00005948) and the National Health Research Authority.

## Results

### Distribution of rubella infection in Zambia

During the 2005–2016 rubella pre-vaccination period, a total of 1313 cases of rubella were confirmed and notified on the basis of IgM sero-positivity in sera of 4497 suspected cases of measles found seronegative for IgM antibodies to measles. The annual cumulative cases of infection ranged from 190 cases in 2005 to 29 cases 2016 albeit high variation of annual reported cases within the period (Table 2). Also important to note is that 2016 was up to the month of September.

**Table 2.** Distribution of new rubella cases per year

Year	Minimum monthly cases	Maximum monthly cases	Median rubella cases	Total rubella cases per annum	Minimum-test positivity rate (%)	Maximum test positivity rate (%)	Median positivity rate for the year (%)
2005	0	45	11	190	0.00	17.90	5.00
2006	0	20	4.5	72	0.00	27.78	4.86
2007	0	41	3.5	101	0.00	40.59	3.48
2008	4	51	13.5	255	1.57	20.00	3.96
2009	0	17	5	76	0.00	22.37	6.58
2010	0	4	1	16	0.00	25.00	6.25
2011	0	30	3	62	0.00	48.39	4.84
2012	1	31	10	150	0.67	20.67	6.67
2013	1	43	9	177	0.57	18.64	5.08
2014	1	21	7	87	1.15	24.14	9.02
2015	2	30	5.5	98	2.04	30.61	5.61
2016	0	14	1	29	0.00	48.28	3.45
Total				Total = 1313	Avg = 0.50	Avg = 28.70	Avg = 5.40

Avg, average.

The highest annual number of 255 was recorded in 2008. The median rubella cases was 5.25 (interquartile range, IQR = 3.13–9.75). However, 2014 recorded the highest median positivity rate of 9.02%.

### Seasonality analysis

**Fig. 2.**
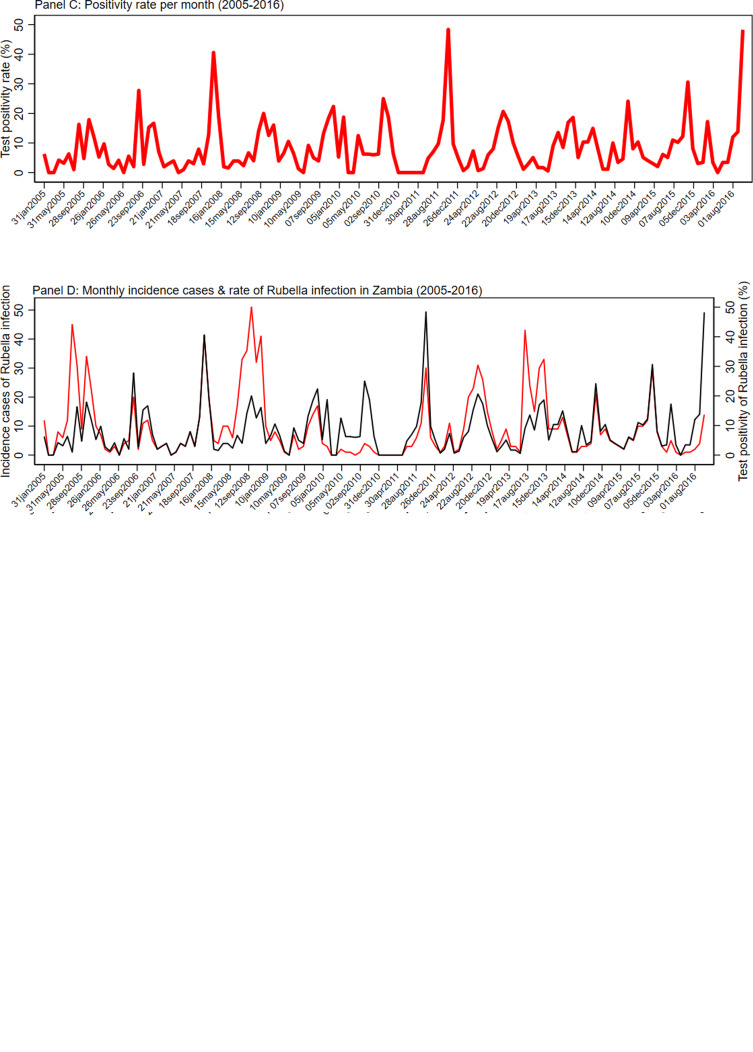
Seasonality pattern of laboratory confirmed rubella. (a) Monthly incidence of rubella infection in Zambia between 2005 and 2016. (b) Cumulative distribution of rubella cases in Zambia between 2005 and 2016. (c) The test positivity rate of rubella infection in Zambia between 2005 and 2016. (d) Monthly distribution of rubella cases (red) and rate of rubella infection between 2005 and 2016.

Figure 2 shows the detailed monthly distribution of cases, cumulative cases and test positivity rate of rubella infection. There was a seasonal pattern in the occurrence of laboratory-confirmed rubella, with peaks in September observed. Although there were significant variations in the monthly test positivity rates in the pre-vaccination period of 2005–2016, higher test positivity rates of rubella infection were usually recorded in the months of September, October and November of each year. The monthly distribution of incidence cases and test positivity rate showed that the highest number of cases and test positivity rate were recorded in the month of September 2008 (51 cases) and October 2011 (48.39%), respectively. Figure 2 details the seasonality pattern of laboratory-confirmed rubella where panel (a) is the monthly incidence of rubella infection in Zambia between 2005 and 2016; panel (b) the cumulative distribution of rubella cases in Zambia between 2005 and 2016; panel (c) the test positivity rate of rubella infection in Zambia between 2005 and 2016; and panel (d) the monthly distribution of rubella cases (red) and rate of rubella infection between 2005 and 2016.

### Forecasting incidence of rubella

**Fig. 3.**
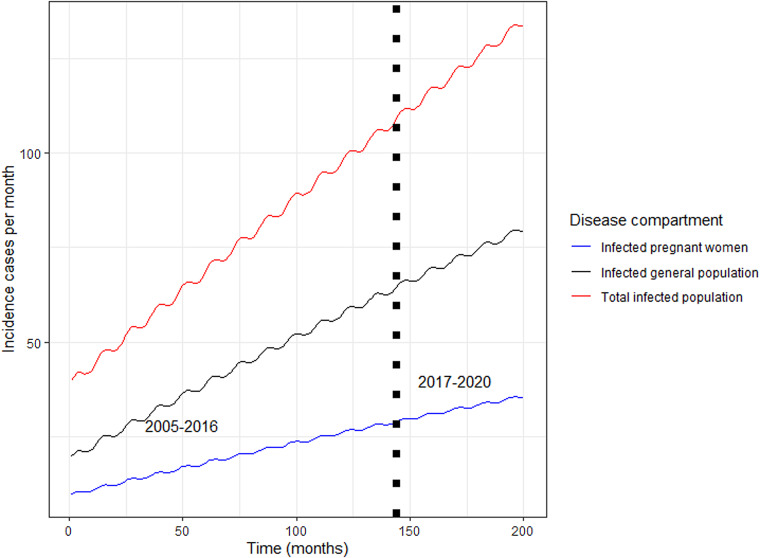
Modelled incidence of rubella cases among the general population and pregnant women in Zambia. *Note*: The vertical dark-short dashed line represents the years before and after vaccine introduction

Figure 3 shows the dynamics of modelled expected incidence of rubella cases among the general population and pregnant women in Zambia. The vertical dark-short dashed line in Figure 3 represents the years before and after vaccine introduction. The modelled monthly median incidence of rubella infection among the general population before vaccination (2005–2016) in Zambia was 76 (IQR = 58–93) and 20 (IQR = 16–25) among pregnant women. The incidence of rubella among the other population (non-pregnant) category was 44 (IQR = 32–53). In the counterfactual scenario, that is assuming no vaccination after 2016, the total expected number of rubella infection among the general population was 89 and 24 among pregnant women. The number of rubella cases among the other population (non-pregnant) category post-vaccination was 53 (IQR = 37–67). The median rate of increase was 0.72 cases per month (95% CI 0.24–1.21, *P* < 0.001).

### Analysis of the effective reproductive number

We explored the trend of effective reproduction number Rt prior to vaccination (2005 and 2016). The intensity of transmissibility and the effectiveness of interventions can be measured by the time-dependent reproduction number Rt, which is the average number of secondary cases caused by an infected individual.

**Fig. 4.**
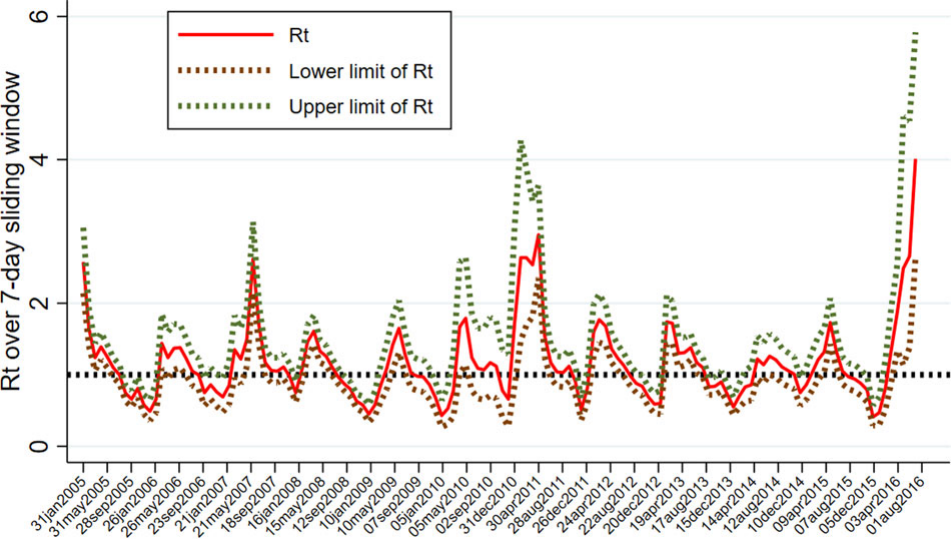
Trend analysis of time-dependent reproduction number (Rt).

Rt initially decreased from an initial median value of 2.6 (95% credible interval (CI) 2.1–3.1) from January 2005 to 0.5 (95% CI 0.4–0.7) in December 2005, and then increased to 1.4 (95% CI 1.1–1.9) in February 2006. The Rt fluctuated above and below the threshold of 1 over the period (2007–2016) but increased to 4.0 (95% CI 2.6–5.8) in June 2016. The annual mean estimates of Rt showed that the lowest Rt of 1.0 was recorded in 2014 (95% CI 0.8–1.1) and the epidemic recorded its peak in 2016 (Rt = 2.2; 95% CI 1.0–3.4). Figure 4 shows the trend analysis of the effective time-dependent reproduction number between 2005 and 2016. In this period there are 12 rubella epidemics.

## Discussion

This is the first study to have modelled the incidence of rubella in Zambia. A total of 1313 cases of rubella were confirmed and notified between 2005 and 2016 with the highest number reported in 2008 (255). The highest median positivity rate in Zambia of 9.02% was recorded in 2014. In the same year 2014, many countries were experiencing increased transmission of rubella with 33 068 active rubella cases reported among 161 countries. Of these 44 were African countries [16].

The modelled monthly median incidence in the general population of 76 was greater than that of pregnant women (20) and non-pregnant women (44). These findings indicate that interventions targeted at the general population would produce the biggest impact on rubella control. The modelled monthly median incidence rate greater than observed value, suggesting underestimates in burden of rubella in general population, partly because the measles surveillance largely covers children under 15 years of age presenting with fever and rash typical of measles infection.

A seasonal pattern of rubella was observed and peaked in September (51 cases). Significant variations in the monthly test positivity rates were detected with higher rates observed in the months of September, October and November with the highest in October (48.4%). Similar variations with a peak in October were observed by Mazaba *et al*., although the peak period in their publication started in July through to November. [17]. A study looking at the epidemiology of rubella in Cameroon between 2008 and 2014 confirms a seasonality pattern with the peaks in February to April was highest, which represents the end of the dry season and beginning of the raining season in most parts of Cameroon. Meanwhile, Kenya in 2014 reports peaks in May, July and October, months characterised with rainy season and cold weather while Zimbabwe in a study between 2007 and 2011 reports peaks in October and November when it is generally dry, a pattern similar to Zambia [18–20]. Rubella usually occurs in a seasonal pattern with highly variable extent and periodicity of the epidemic [1]. In the current study, a similar finding was observed with the epidemic peaking in late September and highest monthly test positivity rate occurring a month later in October. The implication of this finding is that mass vaccination campaigns should be conducted during the period when the incidence rate is at its low levels between January and June.

The annual mean estimates of Rt were between 0.5 and 2 for most of the times except in January 2005 and 2007 and April 2011 and 2016 when Rt was greater than 2. This finding is comparable to findings by Papadopoulos and Vynnycky [12] who estimated the basic reproduction number (Ro) for rubella for 98 settings from around the world and found Ro was <5 for 81 out of 91 settings. In Africa, Ro was in the range 1.5–2.0 between 1979 and 2009. Particularly, in Zambia, the estimate was for the period 1979 and 1980. The finding from the current study suggests that there were 12 rubella epidemics in Zambia between 2005 and 2016. It is expected that the number of epidemics will decrease after the introduction of the vaccine.

Due to paucity in the data, some parameters used in the modelling were obtained from literature which may not be an accurate representation of the rubella epidemiology in Zambia.

In conclusion, the measles-based surveillance system underestimates the observed burden of rubella. The seasonal pattern observed and modelled are consistent. A greater impact of mass vaccination campaign on burden of rubella can occur when conducted among the general population between January and July when cases of rubella are at low levels. It would be of much relevance to re-model the incidence of rubella to date, 6 years after the introduction of routine measles-rubella vaccination in Zambia.

## Data Availability

The dataset on which this paper is based is too large to be retained or publicly archived with available resources. Documentation and methods used to support this study are available from the corresponding author.
